# 6,6′-Dimethyl-2,2′-[1,3-diazinane-1,3-diyl­bis(methyl­ene)]diphenol

**DOI:** 10.1107/S1600536812005284

**Published:** 2012-02-17

**Authors:** Augusto Rivera, Derly Marcela González, Jaime Ríos-Motta, Karla Fejfarová, Michal Dušek

**Affiliations:** a Departamento de Química, Universidad Nacional de Colombia, Ciudad Universitaria, Bogotá, Colombia; b Institute of Physics ASCR, v.v.i., Na Slovance 2, 182 21 Prague 8, Czech Republic

## Abstract

In the mol­ecule of the title compound, C_20_H_26_N_2_O_2_, the 1,3-diazinane ring adopts a slightly distorted chair conformation and the hy­droxy­benzyl substituents occupy equatorial positions on the N atoms of the heterocyclic ring. There are two intra­molecular O—H⋯N hydrogen bonds between the N atoms of the 1,3-diazinane ring and the hy­droxy groups of the aromatic rings, with an *S*(6) set-graph motif. However, the two observed intra­molecular hydrogen-bond distances were different. Considering that both N atoms experience the same chemical environment, it is surprising to see the difference in O⋯N distances [2.6771 (14) and 2.8123 (12) Å]. The crystal structure is further stabilized by a C—H⋯π interaction.

## Related literature

For a previous determination of a related structure, see: Rivera *et al.* (2012 ▶). For a related di-Mannich base, see: Rivera *et al.* (2009 ▶). For the synthesis of the precursor, see: Rivera *et al.* (2010 ▶). For bond-length data, see: Allen *et al.* (1987 ▶). For Cremer–Pople puckering parameters, see: Cremer & Pople (1975 ▶). For hydrogen-bond graph-set nomenclature, see: Bernstein *et al.* (1995 ▶). For the background to hydrogen-bond energy in Mannich bases, see: Koll *et al.* (2006 ▶).
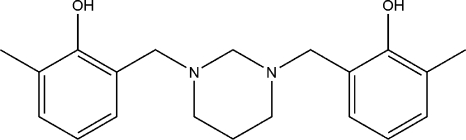


## Experimental

### 

#### Crystal data

C_20_H_26_N_2_O_2_
*M* *_r_* = 326.4
Monoclinic, 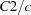

*a* = 31.2788 (5) Å
*b* = 9.7215 (1) Å
*c* = 12.4508 (2) Å
β = 107.936 (2)°
*V* = 3602.00 (10) Å^3^
*Z* = 8
Cu *K*α radiation
μ = 0.62 mm^−1^
*T* = 120 K
0.3 × 0.14 × 0.07 mm

#### Data collection

Agilent Xcalibur diffractometer with an Atlas (Gemini ultra Cu) detector
Absorption correction: multi-scan (*CrysAlis PRO*; Agilent, 2010 ▶) *T*_min_ = 0.615, *T*_max_ = 1
20724 measured reflections
3210 independent reflections
2750 reflections with *I* > 3σ(*I*)
*R*_int_ = 0.031

#### Refinement

*R*[*F* ^2^ > 2σ(*F* ^2^)] = 0.034
*wR*(*F* ^2^) = 0.100
*S* = 1.61
3210 reflections
224 parameters
H atoms treated by a mixture of independent and constrained refinement
Δρ_max_ = 0.15 e Å^−3^
Δρ_min_ = −0.14 e Å^−3^

### 

Data collection: *CrysAlis PRO* (Agilent, 2010 ▶); cell refinement: *CrysAlis PRO*; data reduction: *CrysAlis PRO*; program(s) used to solve structure: *SIR2002* (Burla *et al.*, 2003 ▶); program(s) used to refine structure: *JANA2006* (Petříček *et al.*, 2006 ▶); molecular graphics: *DIAMOND* (Brandenburg & Putz, 2005 ▶); software used to prepare material for publication: *JANA2006*.

## Supplementary Material

Crystal structure: contains datablock(s) global, I. DOI: 10.1107/S1600536812005284/nk2137sup1.cif

Structure factors: contains datablock(s) I. DOI: 10.1107/S1600536812005284/nk2137Isup2.hkl

Supplementary material file. DOI: 10.1107/S1600536812005284/nk2137Isup3.cml

Additional supplementary materials:  crystallographic information; 3D view; checkCIF report

## Figures and Tables

**Table 1:** Hydrogen-bond geometry (Å, °) *Cg*2 is the centroid of the C6–C11 aromatic ring.

*D*—H⋯*A*	*D*—H	H⋯*A*	*D*⋯*A*	*D*—H⋯*A*
O1—H1⋯N1	0.910 (18)	1.818 (19)	2.6771 (14)	156.3 (16)
O2—H2⋯N2	0.898 (16)	2.013 (18)	2.8123 (12)	147.6 (17)
C17—H17⋯*Cg*2^i^	0.96	2.73	3.5577 (14)	144

Symmetry code: (i) 
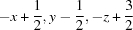
.

**Fig. 1.**
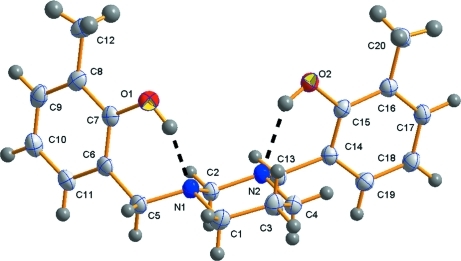
A view of (I) with the numbering scheme. Displacement ellipsoids are drawn at the 50% probability level and H atoms are shown as small spheres of arbitrary radii. Dashed lines indicate intramolecular hydrogen bonds.

## supplementary crystallographic information

### Comment

*Di*-Mannich bases offer convenient models for studying the nature of hydrogen bonding and other weak noncovalent interactions, which play a key role in biological systems. If the OH groups are in appropriate positions relative to the nitrogen lone pairs, both intramolecular and intermolecular hydrogen bonds may be possible. As a rule, non-bonded forms are not observed in systems with phenolic acids that are able to form intramolecular hydrogen bonds, both in solution and in the gas phase (Koll *et al.* 2006).

The molecular structure and atom-numbering scheme are shown in Fig. 1. The C7—O1 and C15—O2 bond lengths [1.3704 (15) and 1.3701 (16) Å, respectively] were comparable with other previously reported C—O bond lengths for *di*-Mannich bases [C—O = 1.365 (2) Å] (Rivera *et al.*, 2009) and [C—O = 1.3762 (11) Å] (Rivera *et al.*, 2012). The C—C bond distances and angles of both aromatic rings were found to be normal (Allen *et al.*, 1987). The CH_2_—N bond lengths, [1.4619 (16) Å and 1.4591 (13) Å] are comparable to the related structure [CH_2_—N = 1.478 (2) Å] (Rivera, *et al.* 2012) but were shorter than the observed lengths in related structure where the heterocyclic ring is the five members: [CH_2_—N = 1.485 (2) Å] (Rivera *et al.*, 2009). In the title compound (I), the 1,3-diazinane ring adopts a slightly distorted chair conformation (Cremer & Pople, 1975) with puckering parameters Q, θ, and φ of 0.5815 (13) Å, 2.32 (13)°, and 91 (3)°, respectively. The molecular structure showed two intramolecular O—H···N hydrogen bonding interactions between the two N atoms and the hydroxyl groups with S(6) set graph motif. However, the two observed intramolecular hydrogen bond distances were different (Table 1). Considering that both nitrogen atoms experiencing the same chemical environment, it is then surprising to see the difference in O···N bond distances ([O1···N1 = 2.6771 (14) Å] and [O2···N2 = 2.8123 (12) Å]).

The hydroxybenzyl substituents occupy equatorial positions. However, the dihedral angles between the mean planes of the benzene rings and the mean plane of the heterocyclic ring were 77.80 (15)° and 80.03 (10)°. This observation indicates there was different spatial positioning, which is more evident with the dihedral angle between both phenyl rings. The angle between the two aromatic rings is 58.431 (38)°.

### Experimental

A solution of 1,3,7,9,13,15,19,21-octaazapentacyclo[19.3.1.^13,7^.1^9,13^.1^15,19^] octacosane prepared according to a previous report (Rivera *et al.*, 2010) (200 mg, 0.54 mmol) in 96% ethanol (5 ml) was added slowly to a stirred solution of 2-methylphenol (240 mg, 2.2 mmol) in 96% ethanol (5 ml) that was heated under reflux. Upon completion of the addition, the reaction mixture was stirred under reflux for 20 h. Next, the reflux was stopped, the solvent was removed on a rotary evaporator under vacuum, and the residue obtained was chromatographed on silica gel eluting with benzene/AcOEt (gradient elution with 5% to 20% AcOEt) to produce a solid which was recrystallized in 96% ethanol to provide high quality crystals of the title compound (I), (Yield 24.4%, m.p. 389–392 K)

### Refinement

All hydrogen atoms were discernible in difference Fourier maps and could be refined to reasonable geometry. According to common practice H atoms bonded C atoms were kept in ideal positions with C–H distance 0.96 Å during the refinement. The methyl H atoms were allowed to rotate freely about the adjacent C—C bonds. The hydroxyl H atoms were found in difference Fourier maps and their coordinates were refined freely. All H atoms were refined with thermal displacement coefficients *U*_iso_(H) set to 1.5Ueq(C, O) for methyl and hydroxyl groups and to to 1.2Ueq(C) for the CH– and CH_2_– groups.

### Crystal data

**Table:**

C_20_H_26_N_2_O_2_	*F*(000) = 1408
*M**_r_* = 326.4	*D*_x_ = 1.204 Mg m^−^^3^
Monoclinic, *C*2/*c*	Cu *K*α radiation, λ = 1.5418 Å
Hall symbol: -C 2yc	Cell parameters from 10886 reflections
*a* = 31.2788 (5) Å	θ = 3.0–67.0°
*b* = 9.7215 (1) Å	µ = 0.62 mm^−^^1^
*c* = 12.4508 (2) Å	*T* = 120 K
β = 107.936 (2)°	Prism, colourless
*V* = 3602.00 (10) Å^3^	0.3 × 0.14 × 0.07 mm
*Z* = 8	

### Data collection

**Table:**

Agilent Xcalibur diffractometer with an Atlas (Gemini ultra Cu) detector	3210 independent reflections
Radiation source: Enhance Ultra (Cu) X-ray Source	2750 reflections with *I* > 3σ(*I*)
Mirror monochromator	*R*_int_ = 0.031
Detector resolution: 10.3784 pixels mm^-1^	θ_max_ = 67.1°, θ_min_ = 3.0°
Rotation method data acquisition using ω scans	*h* = −36→37
Absorption correction: multi-scan (*CrysAlis PRO*; Agilent, 2010)	*k* = −11→11
*T*_min_ = 0.615, *T*_max_ = 1	*l* = −14→13
20724 measured reflections	

### Refinement

**Table:**

Refinement on *F*^2^	H atoms treated by a mixture of independent and constrained refinement
*R*[*F*^2^ > 2σ(*F*^2^)] = 0.034	Weighting scheme based on measured s.u.'s *w* = 1/(σ^2^(*I*) + 0.0016*I*^2^)
*wR*(*F*^2^) = 0.100	(Δ/σ)_max_ = 0.003
*S* = 1.61	Δρ_max_ = 0.15 e Å^−^^3^
3210 reflections	Δρ_min_ = −0.14 e Å^−^^3^
224 parameters	Extinction correction: B–C type 1 Lorentzian isotropic (Becker & Coppens, 1974)
0 restraints	Extinction coefficient: 900 (300)
98 constraints	

### Special details

**Table:**

Experimental. CrysAlis PRO (Agilent Technologies, 2010) Empirical absorption correction using spherical harmonics, implemented in SCALE3 ABSPACK scaling algorithm.
Refinement. The refinement was carried out against all reflections. The conventional *R*-factor is always based on *F*. The goodness of fit as well as the weighted *R*-factor are based on *F* and *F*^2^ for refinement carried out on *F* and *F*^2^, respectively. The threshold expression is used only for calculating *R*-factors *etc*. and it is not relevant to the choice of reflections for refinement.The program used for refinement, Jana2006, uses the weighting scheme based on the experimental expectations, see _refine_ls_weighting_details, that does not force *S* to be one. Therefore the values of *S* are usually larger than the ones from the *SHELX* program.

### Fractional atomic coordinates and isotropic or equivalent isotropic displacement parameters (Å^2^)

**Table:**

	*x*	*y*	*z*	*U*_iso_*/*U*_eq_	
O1	0.06464 (3)	0.07051 (9)	0.66310 (8)	0.0302 (3)	
O2	0.22184 (3)	−0.05521 (9)	0.85547 (8)	0.0307 (3)	
N1	0.09336 (3)	−0.12594 (9)	0.55120 (8)	0.0253 (3)	
N2	0.17352 (3)	−0.14744 (9)	0.63781 (8)	0.0243 (3)	
C1	0.13773 (4)	−0.09468 (12)	0.54144 (10)	0.0246 (4)	
C2	0.08708 (4)	−0.27558 (12)	0.55476 (12)	0.0312 (4)	
C3	0.12437 (4)	−0.33754 (12)	0.65145 (12)	0.0325 (4)	
C4	0.17000 (4)	−0.29827 (12)	0.64228 (11)	0.0287 (4)	
C5	0.05832 (4)	−0.06437 (12)	0.45500 (11)	0.0277 (4)	
C6	0.05613 (4)	0.08973 (12)	0.46498 (10)	0.0251 (4)	
C7	0.05753 (4)	0.14970 (12)	0.56815 (10)	0.0247 (4)	
C8	0.05113 (4)	0.29077 (12)	0.57775 (11)	0.0285 (4)	
C9	0.04476 (4)	0.37139 (13)	0.48171 (12)	0.0329 (4)	
C10	0.04482 (4)	0.31518 (13)	0.37985 (12)	0.0347 (4)	
C11	0.05025 (4)	0.17415 (13)	0.37139 (11)	0.0307 (4)	
C12	0.05171 (5)	0.35190 (14)	0.68853 (13)	0.0404 (5)	
C13	0.21697 (4)	−0.10533 (12)	0.62478 (10)	0.0274 (4)	
C14	0.25656 (4)	−0.14837 (11)	0.72318 (10)	0.0244 (4)	
C15	0.25775 (4)	−0.11617 (11)	0.83352 (10)	0.0239 (4)	
C16	0.29572 (4)	−0.14483 (11)	0.92540 (10)	0.0254 (4)	
C17	0.33194 (4)	−0.20947 (11)	0.90430 (11)	0.0272 (4)	
C18	0.33074 (4)	−0.24696 (13)	0.79600 (11)	0.0291 (4)	
C19	0.29302 (4)	−0.21597 (12)	0.70615 (11)	0.0278 (4)	
C20	0.29665 (5)	−0.10650 (14)	1.04259 (11)	0.0353 (5)	
H1a	0.140383	−0.134347	0.473167	0.0295*	
H1b	0.14095	0.003083	0.536267	0.0295*	
H2a	0.087705	−0.315591	0.484768	0.0374*	
H2b	0.058548	−0.294608	0.565453	0.0374*	
H3a	0.121489	−0.435894	0.64972	0.039*	
H3b	0.121758	−0.30502	0.72196	0.039*	
H4a	0.193116	−0.33274	0.70673	0.0345*	
H4b	0.173643	−0.337606	0.574906	0.0345*	
H5a	0.029635	−0.103489	0.45048	0.0332*	
H5b	0.064148	−0.087548	0.385863	0.0332*	
H9	0.040193	0.468584	0.48637	0.0395*	
H10	0.04114	0.37325	0.315261	0.0416*	
H11	0.049941	0.134735	0.30046	0.0368*	
H12a	0.033234	0.297621	0.721132	0.0605*	
H12b	0.081968	0.353116	0.73863	0.0605*	
H12c	0.040286	0.44419	0.676862	0.0605*	
H13a	0.217355	−0.007301	0.6158	0.0329*	
H13b	0.219925	−0.143722	0.556384	0.0329*	
H17	0.358424	−0.228617	0.966195	0.0327*	
H18	0.355763	−0.293886	0.783351	0.0349*	
H19	0.292099	−0.241654	0.631023	0.0334*	
H20a	0.272699	−0.152761	1.060976	0.053*	
H20b	0.324928	−0.133316	1.095101	0.053*	
H20c	0.29292	−0.008831	1.046816	0.053*	
H1	0.0756 (5)	−0.0090 (18)	0.6433 (14)	0.0454*	
H2	0.1987 (6)	−0.0618 (17)	0.7916 (16)	0.0461*	

### Atomic displacement parameters (Å^2^)

**Table:**

	*U*^11^	*U*^22^	*U*^33^	*U*^12^	*U*^13^	*U*^23^
O1	0.0322 (5)	0.0307 (5)	0.0310 (5)	−0.0004 (3)	0.0144 (4)	0.0017 (3)
O2	0.0253 (4)	0.0363 (5)	0.0309 (5)	0.0075 (3)	0.0093 (4)	−0.0010 (4)
N1	0.0202 (5)	0.0235 (5)	0.0300 (6)	−0.0009 (4)	0.0046 (4)	0.0006 (4)
N2	0.0197 (5)	0.0255 (5)	0.0262 (5)	0.0013 (3)	0.0051 (4)	0.0026 (4)
C1	0.0215 (6)	0.0258 (6)	0.0251 (6)	0.0006 (4)	0.0051 (5)	0.0010 (4)
C2	0.0264 (6)	0.0243 (6)	0.0404 (7)	−0.0037 (4)	0.0068 (5)	−0.0013 (5)
C3	0.0314 (7)	0.0241 (6)	0.0399 (8)	−0.0020 (5)	0.0080 (5)	0.0042 (5)
C4	0.0288 (6)	0.0253 (6)	0.0298 (7)	0.0035 (4)	0.0057 (5)	0.0017 (5)
C5	0.0206 (6)	0.0274 (6)	0.0314 (7)	−0.0003 (4)	0.0025 (5)	−0.0023 (5)
C6	0.0145 (5)	0.0290 (6)	0.0287 (6)	−0.0006 (4)	0.0022 (4)	−0.0007 (5)
C7	0.0157 (5)	0.0287 (6)	0.0295 (7)	−0.0018 (4)	0.0066 (5)	0.0017 (5)
C8	0.0178 (6)	0.0294 (6)	0.0386 (7)	−0.0017 (4)	0.0089 (5)	−0.0040 (5)
C9	0.0226 (6)	0.0255 (6)	0.0467 (8)	0.0003 (4)	0.0048 (5)	0.0013 (5)
C10	0.0296 (7)	0.0341 (7)	0.0348 (7)	0.0005 (5)	0.0017 (5)	0.0087 (5)
C11	0.0252 (6)	0.0355 (7)	0.0268 (7)	0.0007 (5)	0.0016 (5)	0.0012 (5)
C12	0.0392 (8)	0.0369 (7)	0.0498 (9)	0.0007 (5)	0.0210 (6)	−0.0090 (6)
C13	0.0225 (6)	0.0332 (6)	0.0265 (6)	0.0010 (5)	0.0075 (5)	0.0032 (5)
C14	0.0204 (6)	0.0258 (6)	0.0267 (6)	−0.0002 (4)	0.0068 (5)	0.0021 (4)
C15	0.0221 (6)	0.0217 (5)	0.0291 (6)	0.0008 (4)	0.0097 (5)	0.0010 (4)
C16	0.0251 (6)	0.0226 (6)	0.0278 (6)	−0.0018 (4)	0.0070 (5)	0.0010 (4)
C17	0.0222 (6)	0.0265 (6)	0.0300 (7)	0.0002 (4)	0.0037 (5)	0.0030 (5)
C18	0.0222 (6)	0.0304 (6)	0.0350 (7)	0.0038 (5)	0.0093 (5)	0.0003 (5)
C19	0.0257 (6)	0.0306 (6)	0.0285 (7)	0.0009 (4)	0.0101 (5)	−0.0006 (5)
C20	0.0334 (7)	0.0427 (7)	0.0278 (7)	0.0019 (5)	0.0061 (5)	−0.0009 (5)

### Geometric parameters (Å, º)

**Table:**

O1—C7	1.3704 (15)	C8—C9	1.3918 (19)
O1—H1	0.910 (18)	C8—C12	1.497 (2)
O2—C15	1.3701 (16)	C9—C10	1.382 (2)
O2—H2	0.898 (16)	C9—H9	0.96
N1—C1	1.4619 (16)	C10—C11	1.3895 (18)
N1—C2	1.4703 (15)	C10—H10	0.96
N1—C5	1.4784 (14)	C11—H11	0.96
N2—C1	1.4591 (13)	C12—H12a	0.96
N2—C4	1.4727 (15)	C12—H12b	0.96
N2—C13	1.4753 (16)	C12—H12c	0.96
C1—H1a	0.96	C13—C14	1.5084 (14)
C1—H1b	0.96	C13—H13a	0.96
C2—C3	1.5191 (16)	C13—H13b	0.96
C2—H2a	0.96	C14—C15	1.3983 (18)
C2—H2b	0.96	C14—C19	1.3879 (18)
C3—C4	1.515 (2)	C15—C16	1.4000 (14)
C3—H3a	0.96	C16—C17	1.3894 (18)
C3—H3b	0.96	C16—C20	1.4976 (19)
C4—H4a	0.96	C17—C18	1.3861 (19)
C4—H4b	0.96	C17—H17	0.96
C5—C6	1.5066 (16)	C18—C19	1.3861 (15)
C5—H5a	0.96	C18—H18	0.96
C5—H5b	0.96	C19—H19	0.96
C6—C7	1.3990 (18)	C20—H20a	0.96
C6—C11	1.3903 (18)	C20—H20b	0.96
C7—C8	1.3964 (16)	C20—H20c	0.96
			
C7—O1—H1	102.6 (11)	C9—C8—C12	121.73 (11)
C15—O2—H2	106.3 (13)	C8—C9—C10	121.71 (12)
C1—N1—C2	110.32 (9)	C8—C9—H9	119.1442
C1—N1—C5	109.53 (10)	C10—C9—H9	119.1447
C2—N1—C5	110.63 (8)	C9—C10—C11	119.64 (13)
C1—N2—C4	109.49 (8)	C9—C10—H10	120.1785
C1—N2—C13	108.15 (9)	C11—C10—H10	120.1783
C4—N2—C13	111.25 (9)	C6—C11—C10	120.44 (13)
N1—C1—N2	111.48 (10)	C6—C11—H11	119.78
N1—C1—H1a	109.4714	C10—C11—H11	119.7804
N1—C1—H1b	109.4721	C8—C12—H12a	109.4711
N2—C1—H1a	109.4702	C8—C12—H12b	109.4722
N2—C1—H1b	109.4711	C8—C12—H12c	109.4711
H1a—C1—H1b	107.3846	H12a—C12—H12b	109.4712
N1—C2—C3	109.82 (9)	H12a—C12—H12c	109.471
N1—C2—H2a	109.471	H12b—C12—H12c	109.4707
N1—C2—H2b	109.4726	N2—C13—C14	112.79 (10)
C3—C2—H2a	109.4711	N2—C13—H13a	109.4717
C3—C2—H2b	109.4718	N2—C13—H13b	109.4711
H2a—C2—H2b	109.1181	C14—C13—H13a	109.4713
C2—C3—C4	110.58 (11)	C14—C13—H13b	109.4711
C2—C3—H3a	109.4709	H13a—C13—H13b	105.9345
C2—C3—H3b	109.4706	C13—C14—C15	120.18 (11)
C4—C3—H3a	109.4712	C13—C14—C19	121.00 (11)
C4—C3—H3b	109.4715	C15—C14—C19	118.79 (10)
H3a—C3—H3b	108.3382	O2—C15—C14	121.16 (9)
N2—C4—C3	109.68 (10)	O2—C15—C16	117.68 (11)
N2—C4—H4a	109.4709	C14—C15—C16	121.16 (11)
N2—C4—H4b	109.4709	C15—C16—C17	118.13 (12)
C3—C4—H4a	109.4718	C15—C16—C20	120.24 (11)
C3—C4—H4b	109.4711	C17—C16—C20	121.63 (10)
H4a—C4—H4b	109.2658	C16—C17—C18	121.56 (10)
N1—C5—C6	112.15 (9)	C16—C17—H17	119.2196
N1—C5—H5a	109.4713	C18—C17—H17	119.2211
N1—C5—H5b	109.4705	C17—C18—C19	119.30 (12)
C6—C5—H5a	109.4715	C17—C18—H18	120.3511
C6—C5—H5b	109.4715	C19—C18—H18	120.3499
H5a—C5—H5b	106.6581	C14—C19—C18	120.99 (12)
C5—C6—C7	120.05 (11)	C14—C19—H19	119.5056
C5—C6—C11	121.03 (11)	C18—C19—H19	119.5079
C7—C6—C11	118.83 (11)	C16—C20—H20a	109.4711
O1—C7—C6	120.60 (10)	C16—C20—H20b	109.4716
O1—C7—C8	117.86 (11)	C16—C20—H20c	109.4707
C6—C7—C8	121.54 (11)	H20a—C20—H20b	109.4715
C7—C8—C9	117.77 (12)	H20a—C20—H20c	109.4703
C7—C8—C12	120.50 (12)	H20b—C20—H20c	109.4722

### Hydrogen-bond geometry (Å, º)

*Cg*2 is the centroid of the C6–C11 aromatic ring.

**Table:**

*D*—H···*A*	*D*—H	H···*A*	*D*···*A*	*D*—H···*A*
O1—H1···N1	0.910 (18)	1.818 (19)	2.6771 (14)	156.3 (16)
O2—H2···N2	0.898 (16)	2.013 (18)	2.8123 (12)	147.6 (17)
C17—H17···*Cg*2^i^	0.96	2.73	3.5577 (14)	144

Symmetry code: (i) −*x*+1/2, *y*−1/2, −*z*+3/2.
